# Expanding access to high-cost medicines under the Universal Health Coverage scheme in Thailand: review of current practices and recommendations

**DOI:** 10.1186/s40545-023-00643-z

**Published:** 2023-11-07

**Authors:** Dimple Butani, Dian Faradiba, Saudamini Vishwanath Dabak, Wanrudee Isaranuwatchai, Evan Huang-Ku, Kumaree Pachanee, Budsadee Soboon, Anthony J. Culyer, Yot Teerawattananon

**Affiliations:** 1 Health Intervention and Technology Assessment Program (HITAP), Department of Health, Ministry of Public Health, 6th Floor, 6th Building, Tiwanon Road, Nonthaburi, 11000 Thailand; 2 Saw Swee Hock School of Public Health, National University of Singapore (NUS), 12 Science Drive 2, #10-01, Singapore, 117549 Singapore; 3 Institute of Health Policy, Management and Evaluation, St. Michael’s Hospital, University of Toronto, 30 Bond St, Toronto, ON M5B 1W8 Canada; 4 Dalla Lana School of Public Health, University of Toronto, 155 College St Room 500, Toronto, ON M5T 3M7 Canada; 5 Department of Economics and Related Studies and Centre for Health Economics, University of York, York, UK

**Keywords:** Expensive medicines, Rare disease, Cancer medicines, Managed entry agreement, Reimbursement pathways

## Abstract

**Background:**

There has been an increasing demand to reimburse high-cost medicines, through public health insurance schemes in Thailand.

**Methods:**

A mixed method approach was employed. First, a rapid review of select high-income countries was conducted, followed by expert consultations and an in-depth review of three countries: Australia, England and Republic of Korea to understand reimbursement mechanisms of high-cost medicines. In Thailand, current pathways for reimbursing high-cost medicines reviewed, the potential opportunity cost estimated, and stakeholder consultations were conducted to identify context specific considerations.

**Results:**

High-income countries reviewed have implemented a variety of pathways and mechanisms for reimbursing high-cost medicines under specific eligibility criteria, listing processes, varying cost-effectiveness thresholds and special funding arrangements. In Thailand, high-cost medicines that do not offer good value-for-money are excluded from the reimbursement process. A framework for reimbursing high-cost medicines that are not cost-effective at the current willingness-to-pay threshold was proposed for Thailand. Under this framework, specific criteria are proposed to determine their eligibility for reimbursement such life-saving nature, treatment of conditions with no alternative treatment options, and affordability.

**Conclusion:**

High-cost medicines may become eligible for reimbursement through alternative mechanisms based on specific criteria which depend on each context. The application of HTA methods and processes is important in guiding these decisions to support sustainable access to affordable healthcare in pursuit of Universal Health Coverage (UHC).

**Supplementary Information:**

The online version contains supplementary material available at 10.1186/s40545-023-00643-z.

## Key message

- There is a growing demand for public health insurance programmes to reimburse high-cost medicines, including those that do not represent good value-for-money. This demand is likely to inhibit financial sustainability, given the growth of new technologies and public demands for expanded access to care.
- This study reviewed the experience of selected countries with established health technology assessment (HTA) systems and found that almost all have initiated special programmes or alternative mechanisms to fund high-cost, cost-ineffective medicines as they progress towards Universal Health Coverage (UHC).
- We recommend no reimbursement for cost-ineffective medicines and reimbursement of other high-cost medicines only when they are the only life-saving option available to patients.
- In such cases, contextual factors may be taken into account to determine the appropriate willingness-to-pay threshold and budget limits. Monitoring and evaluation of implementation arrangements will be critical for assessing the impact of these programmes.

**Supplementary Information:**

The online version contains supplementary material available at 10.1186/s40545-023-00643-z.

## Background

Recent scientific advancements have resulted in the development of several novel medical interventions that offer to improve the quality and length of life for patients with life-threatening conditions. These novel medical interventions include gene therapies, tissue-engineered medicines, and somatic-cell therapy targeting various cancer, rare, and ultra-rare diseases. However, the cost of these medicines is extremely high with the top ten most expensive medicines in 2022 ranging from USD 600,000 to over USD 3.5 million in price per dose [1, 2].

The high cost of these medicines results in high out-of-pocket expenses for households requiring such medicines. This reduces, delays, or even denies access to care which could have otherwise save lives or significantly improve people’s quality of life. There are therefore calls for government intervention to ensure accessibility to healthcare services with their commitment to Universal Health Coverage (UHC) [3]. However, governments around the world, including high-income countries, are struggling to meet the increasing demand to reimburse them in their benefits package [4] and are often left with dilemma of whether a “high-cost medicine” is worth paying in view of lower cost treatments it may crowd out. This is particularly concerning for low- and middle-income countries (LMICs) which are even more resource constrained.

Currently, there is no universally agreed upon definition of the term high-cost medicine and it is frequently used to describe medicines whose Incremental Cost-Effectiveness Ratio (ICER) exceeds the public willingness-to-pay threshold for a Quality-Adjusted Life Year (QALY) [5, 6]. Including them in the benefits package of a public health insurance scheme translates into a high opportunity cost to a health system, by virtue of the more productive treatments that no longer become affordable from the available budget. If the budget is increased to enable public purchase, then the opportunity cost becomes general consumption as resources become withdrawn from private consumption via taxation or other means.

Thailand, an upper middle-income country in Southeast Asia, uses Health Technology Assessment (HTA), including cost-effectiveness analysis, to guide the development of its pharmaceutical benefits package, the National List of Essential Medicine (NLEM). The NLEM is an optimum list of medicines referred to as a “reimbursement list” for all three public health insurance schemes in Thailand [7]. It is unlikely for a medicine to be reimbursable outside the NLEM; however, these medicines are available on the market for patients to purchase at out-of-pocket expenses or through additional insurances. Over the years, there has been increasing pressure to include medicines deemed cost-ineffective, and oncologists have submitted medicines outside of the NLEM for reimbursement to the National Health Security Office (NHSO), which manages the largest public health insurance scheme, the Universal Coverage Scheme (UCS).

To understand the potential for reimbursing high-cost medicines that are not currently cost-effective, the NHSO commissioned the Health Intervention and Technology Assessment Program (HITAP), a Thai national HTA agency, to conduct a study to provide recommendations to a Working Group that was set up to advise on the management of such medicines as part of the UCS Benefit Package (UCBP). Given the above, a study was conducted with the following objectives: (1) to understand the landscape of policy process supporting the reimbursement decisions on high-cost medicines in the context of select high-income countries with established HTA systems; (2) to understand their access in Thailand; and lastly (3) to propose a framework for evaluating high-cost medicines for reimbursement under UCS as well as the Thai NLEM, which covers all three major public health insurance schemes, in Thailand.

## Methods

**Fig. 1:**
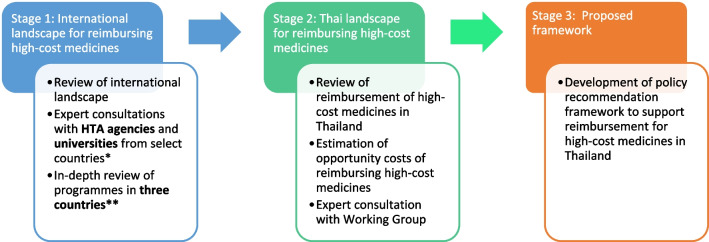
Methods summary. *HTA* Health Technology Assessment. *HTA agencies and Universities from select countries: Singapore: Agency for Care Effectiveness (ACE); Canada: Drug and Health Technology Agency (CADTH); England: National Institute for Health and Care Excellence (NICE) and University of York; Malaysia: Malaysian Health and Technology Assessment (MaHTAS); Republic of Korea: National Evidence-based Healthcare Collaborating Agency (NECA); Australia: Royal Adelaide Hospital **Australia, Republic of Korea, and England

To achieve the three objectives, the study was conducted in two stages, adapting a method for conducting rapid reviews for complex questions [8]. The aim of the first stage was to understand the existing international landscape for reimbursing high-cost medicines. In the second stage, we aimed to understand the current mechanisms and expectations for reimbursing novel high-cost medicines in Thailand. Findings from these two steps were then used to describe the reimbursement mechanisms and thereby propose policy options for expanding access to high-cost medicines in Thailand. Figure 1 summarises the methods used for this study.

### Describing the international landscape for reimbursing high-cost medicines

#### Review of international landscape

A rapid review was conducted to identify relevant published and grey literature on the topic of high-cost medicines. Additionally, the websites of official HTA agencies of selected countries, namely England, Australia, Malaysia, the Republic of Korea (hereafter referred to as “South Korea”), Canada, and Singapore were reviewed (see Additional file 1: Table S1 for a list of countries and references). These countries were purposively chosen as they have established HTA policies and processes to guide the development of their benefit packages [9–12]. Six experts with a background in health economics and significant knowledge or practical experience in medicine reimbursements in the aforementioned select countries were consulted through online stakeholder consultations. This consultation served to verify the collected data and address any remaining gaps. The responses were not informed to other stakeholders (Additional file 1: Table S2).

#### In-depth review

Three countries were selected for an in-depth review based on findings from the initial review to understand the quality and completeness of the literature describing their experience with respective pathways for reimbursing high-cost medicines. These pathways were England’s Cancer Drugs Fund (CDF) and Highly Specialised Technologies (HST) evaluation programme [13, 14], Australia’s Life-Saving Drug Program (LSDP) [15], and South Korea’s New drug pathway [12].

#### Analysis

The findings were reported in the form of a narrative summary of the information collected and organised in terms of the steps of the HTA process covering the following areas: (a) criteria for reimbursement of high-cost medicines; (b) the alternative reimbursement process and pathways; (c) listing process; and (d) monitoring and evaluation of the reimbursement pathways.

### Understanding the current landscape for reimbursing high-cost medicines in Thailand

#### Review of high-cost medicines reimbursement in Thailand

Cancer medicines have significant budget impact [16, 17]. To understand the difference in consideration for inclusion in the NLEM, a comparison was made between the list of cancer medicines submitted to NHSO’s Cancer Working Group and the medicine list submitted to the NLEM from the year 2019 to 2021. This comparison aimed to identify which high-cost cancer medicines were included into the NLEM submitted list; with that additionally, the list of cancer medicines was compared of the World Health Organization (WHO) Model List of Essential Medicines (EML) and the NICE guidance on clinical practice to determine recommended medicines by these organisations. The results were summarised in a three-by-three table, indicating whether the proposed list of medicines was recommended, not-recommended or never considered by the WHO EML, NICE and the NLEM.

#### Estimation of the opportunity cost of including high-cost cancer medicines

The next step involved estimating the trade-off when investing in the cancer medicines submitted for inclusion in Thailand. The authors extracted information from completed studies between 2008–2021 in Thailand that evaluated cost-effectiveness of the cancer medicines for the same or similar indications. Using the ICER reported in the literature, the QALYs lost were calculated as per the formula below, against Thailand’s willingness-to-pay threshold of 160,000 Thai Baht (THB)/QALY [7]:

$$QALYs lost=\left(\frac{budget impact of medicines }{\mathrm{incremental cost of medicines }}\right)-\left(\frac{budget impact of medicines }{willingness to pay threshold}\right).$$

The results of the analysis were summarised in tabular format and presented to stakeholders of the Working Group.

#### Expert consultation with Working Group in Thailand

Following the review, three rounds of expert consultations with the Working Group were conducted to identify the considerations for including high-cost medicines in Thailand. This Working Group was appointed by the payer, i.e. NHSO based on convenience sampling and constituted of 15 experts comprising health policy, clinicians, health economists and other relevant subject matter experts. These online consultations were held from the end of May 2022 to July 2022 with members of Working Group that consisted of representatives from the Thai Food and Drug Administration (FDA), members from the sub-committee for the development of the NLEM and the National List of Essential Vaccines (NLEV) sub-committee, the NHSO, and the price negotiation Working Group of the NLEM, along with other members from the public sector and public funding agencies.

During the initial two rounds of consultations, the Working Group was presented with the findings from the review of international experiences and the existing situation of high-cost cancer medicines in Thailand. Additionally, a survey was conducted among the Working Group members to elicit the information on definition of high-cost medicines and life-saving medicines, criteria for reimbursement, and proposed budget limit for high-cost medicines in Thailand. The survey was administered in Thai using the Survey Sparrow platform, and the results were displayed for review by all participants during the consultative meeting. A translated version is available in English in Additional file 1: Figure S1. The collected data were analysed descriptively and presented in a tabular format.

#### Recommendation framework

Lastly, the authors developed a conceptual framework through an iterative process describing the path for reimbursing high-cost medicines in Thailand. The findings of which were presented further to the Working Group for their feedback.

## Results

The results from the analysis are summarised in three parts: (1) international experience in reimbursing high-cost medicines; (2) current landscape for reimbursing high-cost medicines in Thailand, and (3) policy recommendations for reimbursing high-cost medicines in Thailand.

### International experience in reimbursing high-cost medicines

Key findings from three countries for which an in-depth review was conducted, are summarised below. Additional information on all six countries reviewed is presented in Additional file 1: Table S2.

#### Pathways and criteria for reimbursing high-cost medicines

All the countries reviewed had established alternative mechanisms for reimbursing high-cost medicines and employed a set of eligibility criteria to identify them. These criteria include factors such as disease severity, rarity, the effectiveness of medicines, treatment of a life-threatening condition, and the absence of alternative treatment options for treating the same condition.

In South Korea, for example, new medicines that do not have an alternative treatment option may be reimbursed without providing evidence of cost-effectiveness. A pharmaco-economic waiver (PE waiver) is provided, and the price is set after the National Health Insurance Service (NHIS) which is the payer and a manufacturer enter into some form of contractual risk-sharing arrangement (RSA) [12]. Similarly, Australia’s LSDP provides access to rare disease medications that are either cost-ineffective or deemed to be too expensive. A medicine must meet certain criteria in order to be listed in the LSDP which then allows reimbursement at no cost to patients [15]. High-cost medicines in England are either reimbursed through the CDF or reimbursed under the HST programme that allows for having a different cost-effectiveness threshold [14, 18]. Table 1 summarises the pathways and corresponding eligibility criteria for each country.

**Table 1:** Criteria for high-cost medicines in select three countries

No.	Country and programme/pathway	Criteria
1	Australia Life-Saving Drug Program (LSDP)	1) The medicine should be clinically effective but not sufficiently cost effective to be listed on the Pharmaceutical Benefits Scheme (PBS) 2) the medicine is used to treat life-threatening and rare conditions (defined as 1 case per 50,000 people or less) and 3) if the pharmaceutical company (sponsor) applies for an LSDP listing
2	England reformed Cancer Drugs Fund (CDF) & Highly Specialised Technology (HST)	1) Uncertainty in Incremental Cost-Effectiveness Ratio (ICER) 2) Increase health related quality of life (QoL) 3) Technology innovation 4) Medicine to extend one’s life 5) If indicated for diseases with short life expectancy and 6) Aspects that relate to non-health objectives of NHS such as equity
3	South Korea Pharmaco-economic (PE) waiver	1) There is no alternative treatment 2) The new medicine treats a life-threatening or rare disease and/or cancer 3) Treats small patient group 4) Should have proven clinical efficacy and 5) The medicine should be listed in at least three of the seven A7 countries that Korea refers to for medicine prices (“A7” are seven countries: the United States, the United Kingdom, Italy, Germany, Japan, Switzerland, and France)

#### Listing process

The listing process for high-cost medicines varies across the three countries. To determine whether a medicine meets the aforementioned criteria, the applicant, often the sponsor company, applies to an independent appraisal committee appointed by the government. An appraisal committee assesses the validity of the submitted clinical and cost-effectiveness evidence. During the assessment phase, an evidence group will determine whether the medicine meets the criteria and will draft an evidence-based recommendation. The time for appraisal ranges from 2 to 6 weeks in Australia to 90 days in England [18]. Table 2 summarises the process of listing high-cost medicines in each country.

**Table 2:** Process for listing high-cost medicines in Australia, England, and South Korea

Process for listing high-cost medicines	Australia	England	South Korea
*Responsible parties in each step for listing of high-cost medicines*			
Submit the application	Sponsor* company submits a dossier after medicine is rejected by PBS	All novel cancer medicines submitted by sponsor	Application and related dossier submitted by sponsor company
Review the evidence	LSDP expert panel	NICE appraisal committee	HIRA—BCA committee and EE sub-Committee
Appraise the evidence	Chief Medical Officer (LSDP)	NICE advisory committee	PBC
Time for appraisal	2–6 weeks	90 days	120 days
Price Negotiations	MoH and Sponsor	NHS and Sponsor	NHIS and Sponsor
Final decision	MoH	NICE	MOHW
Re-evaluation	MoH every 2 years	NICE every 2 years	MOHW every 2 years

*BCA* Benefit Criteria Advisory, *EE* economic evaluation, *HIRA* Health Insurance Review and Assessment Service, *LSDP* Life Saving Drug Program, *MoH* Ministry of Health, *MOHW* Ministry of Health and Family Welfare, *NICE* National Institute for Health and Care Excellence, *NHIS* National Health Insurance Service, *PBC* Pharmaceutical Benefits Committee

*Sponsor: a pharmaceutical manufacturer or any company that nominates a medicine for listing

An advisory committee makes the final decision for reimbursement. However, an agreement is signed only if the sponsor company agrees to the conditions stated in the appraisal committee's final dossier. The final negotiation takes place with the pharmaceutical company and the funding agency of respective country like NHS for England, or PBS for Australia and for NHIS for South Korea. Lastly, medicines listed for reimbursement are evaluated by an evaluation committee to assess the appropriateness of the listing decision and a decision-maker, who is a government authority, makes the final decision to enact the recommendation or otherwise. Having a special pathway allows for a fast-track approval of high-cost new medicines. For instance, in the case of NICE, a medicine can be made available within 60 calendar days after the publication of the appraisal committee’s report.

#### Special funding arrangements

Several countries, including England, Australia, and South Korea, established special funds to provide financial assistance to patients requiring high-cost medicines. The CDF programme was established in 2010 to provide patients with access to cancer medicines that were not routinely available as part of the NHS, with a budget limit of British Pound Sterling (GBP) 200 million. To address the issues of sustainability and transparency, the CDF underwent significant reforms in 2016, involving implementation of a new medicines review process and a cap on the amount of money (GBP 340 million) that could be spent on the programme. Similar to CDF, an Innovative Medicines Fund (IMF) [18, 19] was launched to address the limitations of the CDF which prioritises only cancer medicines. In Australia, the LSDP [15] provides access to high-cost medicines, whereas South Korea has a special funding programme called the "High-Cost Rare Disease Treatment Support Program” for rare disease treatments not covered by the National Health Insurance Service (NHIS) [20]. The amount allocated to either programme is subject to change based on factors such as the cost of new and existing medicines, changes in demand, and the overall state of the economy.

#### Special cost-effectiveness threshold

There is a general trend towards having a higher cost-effectiveness threshold for innovative medicines that are expensive. In England, for example, the new HST programme considers assigning additional weights to QALYs above the most plausible ICER of GBP 100,000 per QALY gained, and the committee considers assigning additional weights to QALYs for a technology whose ICER rises up to GBP 300,000 per QALY gained [14]. Typically, the cost-effectiveness threshold is not explicitly stated; however, elasticity to the upper limit of ICER is applicable under certain conditions; for example, in Australia, cost-ineffective medicines treating severe and progressive diseases affecting a small number of patients when there is no existing alternative treatment are reimbursed [21]. The Introduction of the Benefit Enhancement Plan (IBEP) in South Korea allowed for an increase in the cost-effectiveness threshold for medicines for cancer, cardiovascular, cerebrovascular, and rare diseases with no alternatives; however, this increase in threshold is kept confidential.

#### Managed entry agreements (MEAs)

MEAs, also known as risk-sharing agreements (RSA), managed access agreements (MAA), and patient access schemes (PAS) [22], are essentially contractual agreements between payers and manufacturers that are subject to pre-defined conditions [22, 23]. MEAs are often used as a mechanism to reduce the risk of uncertainty in budget impact, clinical and cost-effectiveness. Broadly, MEAs can be categorised into health outcome-based and financial-based agreements [24]. Outcome-based agreements, also known as performance-based agreements, involve assessing effectiveness through the collection of clinical data. This evaluation is often based on a surrogate outcome related with the endpoint of interest or by the endpoint itself, with adjustments made to price accordingly. In contrast, financial-based contracts, utilises discounts/refunds, price expenditure caps, or volume-based caps are and are more commonly used in countries than outcome-based MEA’s. This is mainly due to several administrative challenges, with outcome-based agreement specifically regarding data collection and confidentiality. These agreements require tracking specific endpoints or desired outcomes, necessitating access to sensitive information such as patient records, clinical trials or other propriety data, safeguarding of which can become add to administrative complexities in managing such agreements. Table 3 summarises the various types of MEAs being implemented in the countries reviewed.

**Table 3:** Summary of types of MEAs implemented in select countries

Country	MEA used	Medicines reimbursed
Australia	The risk-sharing arrangement is captured through a legal deed of agreement (‘deed’) that is negotiated between the sponsor and the government. Some financial risk share arrangements can be class deeds where sponsors share the risk based on market share	A financial risk share was mentioned for 24 medicines in the most recent public summary documents
England	NHS and manufacturers have an agreement and one of the functions of CDF is managed access fund providing conditional funding for cancer medicines where uncertainty is addressed through data collection. Dominantly financial MEAs in form of discounts are used, but outcome-based MEA is also performed	England has approved 42 medicines since the introduction of the CDF
South Korea	Four types of MEAs: (i) coverage with additional evidence, (ii) expenditure cap refund, (iii) utilisation cap per patient and (iv) refund/expenditure cap	As of 2019, 39 medicines had been reimbursed under RSA

*CDF* Cancer Drugs Fund, *MEA* Managed Entry Agreement, *NHS* National Health Service, *RSA* Risk Sharing Agreement

## Monitoring and evaluation (M&E)

The role of M&E is to assess the safety and effectiveness of the medicine, and whether medicine prices are justified based on their performance. Across the three countries reviewed, typically, the evaluation begins 24–48 months after the medicine is listed. However, data collection, including patient-level data, starts as soon as a medicine is listed.

In Australia, M&E is overseen by the PBAC and LSDP expert panels. These panels review additional documents, such as clinical effectiveness evidence and international evidence, to support their final recommendations to the government. These recommendations may include changing eligibility criteria or treatment guidelines, amendments to existing MEAs, termination of medicine reimbursed, negotiation of lower price, or requirement for additional data collection from LSDP listing.

Clinical evidence is gathered in England throughout the duration of the conditional agreement scheme, which usually lasts 24 months. NICE conducts regular reviews on the uncertainty of approved medicines during the contractual agreements period. After the agreement expires, NICE provides recommendations on whether continued treatment for the same indication should be recommended or discontinued based on cost-effectiveness evidence. South Korea conducts regular audits for medicines approved under the pharmaco-economic (PE) waiver or listed through the MEA pathway. Generally, medicines listed through an MEA requires submission of evidence of effectiveness (such as lack of alternative treatment, improved of survival and quality of life) every 4 years to maintain eligibility for extension of exemption.

### Reimbursing high-cost medicines in Thailand

#### Identification of proposed high-cost medicines included in the NLEM

During the period of 2019–2021, a total of 43 cancer medicines were submitted for consideration to inclusion in the NLEM. Out of these, only 18 medicines were proposed for consideration into NLEM for further evaluation. Among the submissions, ten medicines were rejected due to a lack of supporting evidence regarding their effectiveness based on six criteria: efficacy, safety, disease burden, evidence of economic evaluation, budget impact, and social support [7]. On the other hand, 16 medicines were never submitted for evaluation because of the expected high-cost and were deemed not cost-effective at the threshold value of 160,000/QALY.

#### Comparison of proposed high-cost medicines with WHO EML and NICE guidelines

A total of 43 cancer medicines for 63 indications, which were proposed for consideration to NLEM, were compared in relation to recommendations provided by the WHO EML and NICE clinical guideline. The analysis found that: (i) 20 of these medicines were included in the NICE clinical guidance; (ii) seven medicines were included in the WHO EML; (iii) four medicines were included in both, the NICE clinical guidance and WHO EML and (iv) there were two medicines for three specific indications that were neither recommended by NICE or the WHO EML but were included in Thailand’s NLEM. A summary of these findings can be found in Additional file 1: Fig. S2.

#### Review of the opportunity cost of including high-cost medicines in the NLEM

The opportunity cost associated with the introduction of the proposed high-cost medicines was estimated by utilising data from cost-effectiveness studies for five medicines that were already included in the NLEM. The results are summarised in Table 4, indicating that medicines that are not good value-for-money (cost-ineffective) can lead to a substantial QALY loss. To illustrate this, consider the example of lenalidomide, a high-cost medicine indicated for patients with plasma cell leukaemia. Its ICER value was determined to be THB 12,009,328/year. If lenalidomide were to be reimbursed, the ensuring opportunity cost for reimbursing it would lead to 1031 QALYs, calculated at the THB 160,000/QALY threshold value [7]. Leuprorelin, on the other hand, demonstrated positive QALY gains at the designated threshold value, leading to its inclusion in the NLEM [25].

**Table 4:** Incremental cost-effectiveness ratio of 5 medicines from NLEM compare with budget and QALYs loss

Medicines (of the 43 medicines that had economic evaluation)	Indication	ICER	Budget (THB/year)	QALYs gained (A)	QALYs gained at WTP (B)	QALYs lost (A-B)
Leuprorelin	Prostate cancer with intermediate risk	137,613	351,000,000	2551	2194	357
Sorafenib	Metastatic clear cell renal cell carcinoma	1,650,000	18,576,000	11	116	− 105
Bortezomib	Multiple myeloma	9,908,461	119,200,000	12	745	− 733
Thalidomide	Multiple myeloma	10,706,411	94,400,000	9	590	− 581
Lenalidomide	Multiple myeloma	12,009,328	167,200,000	14	1045	− 1031

*ICER* incremental cost-effectiveness ratio, *QALY* Quality-Adjusted Life Year, *WTP* willingness to pay

1 USD = 34.97 THB as of July, 2023

#### Expert consultation of Working Group

The findings from our international review and assessment of the national context informed a survey that was distributed among stakeholders of the Working Group. The survey aimed to gather insights on the definition and reimbursement criteria for high-cost medicines and life-saving medicines. Regarding the definition, the Working Group voted that any medicinal therapeutic intervention without viable treatment alternatives, costing above THB 1–2 million per medicine or per dose per year, would be categorised as high-cost. In terms of defining life-saving medicines, specific criteria were agreed: (a) the medicine prolongs lifespan by at least 1 year, (b) the medicine prevents severe co-morbidities, (c) the medicine significantly improves quality of life and (d) failure to administer the medicine within 6 months would lead to death. Based on these criteria, it was recommended that a high-cost medicine meeting the defined conditions ought to be made available under the three public health insurance schemes. However, during the initial 2–3 years of implementation, a budget limit of THB 500 million per year (USD 1.5 million; 1 USD = 34.97 THB as of July, 2023), which is three times the current amount spent, was suggested.

### Proposed mechanism for reimbursing high-cost medicines in Thailand

**Fig. 2:**
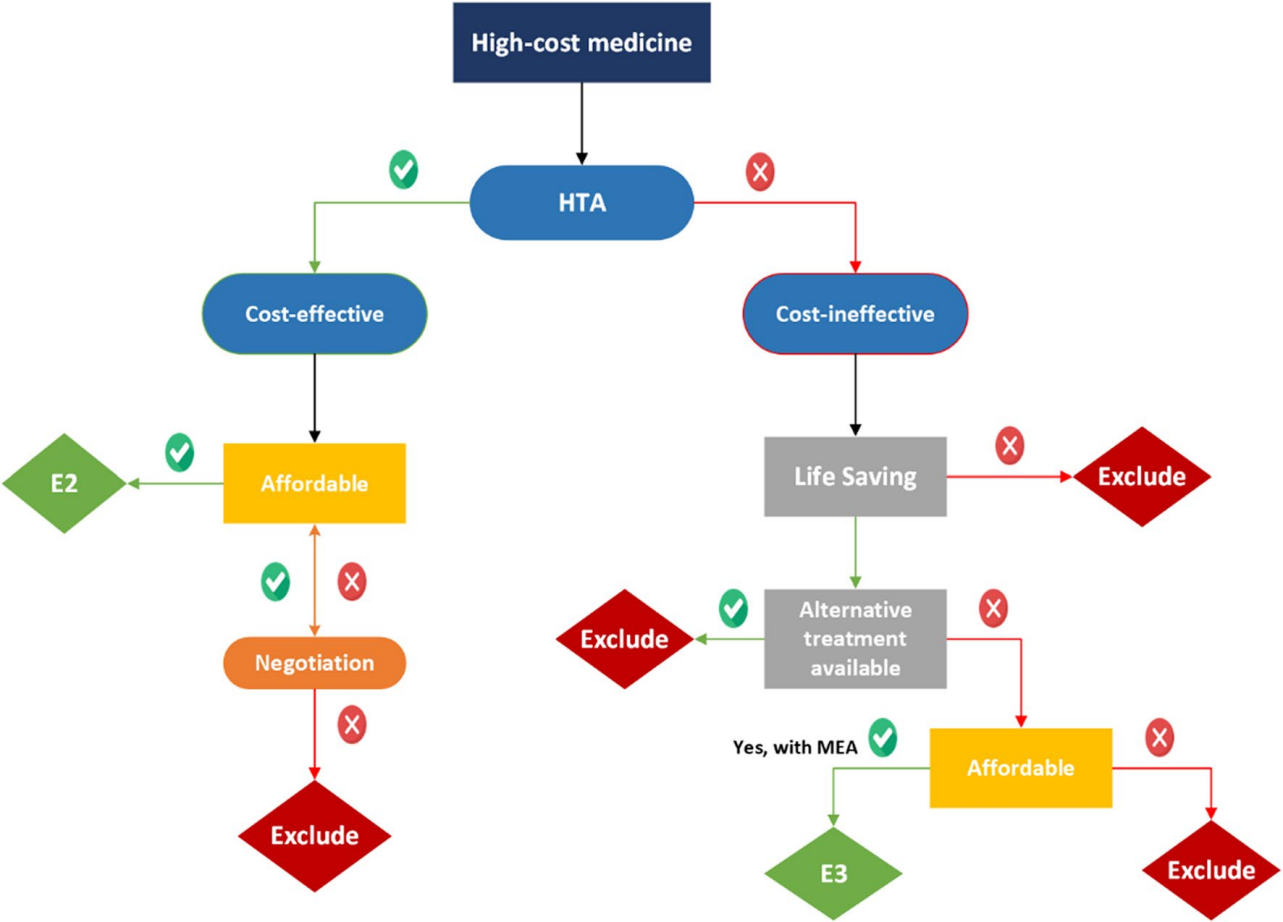
Proposed framework for reimbursing high-cost medicines in Thailand. E2, medicines that are of high cost but are important for groups of patients; E3, proposed category to reimburse life-saving high-cost medicines with no alternative treatment; HTA, Health Technology Assessment; MEA, Managed Entry Access

A framework was developed to guide the reimbursement of high-cost medicines in Thailand. This framework proposes creation of a new sub-category, “E3” to the NLEM to access high-cost medicine under the benefit package (Fig. 2). Under this “E3” category, if a medicine is deemed cost-ineffective based on evidence of economic evaluation, these medicines may be considered for reimbursement only if they are lifesaving and there are no available alternative treatments. Affordability, in terms of budget impact, would be taken into account by the sub-committee (final decision-making body) based on the abovementioned criteria for inclusion into NLEM. If a medicine meets these criteria, despite being cost ineffective, it may be considered for inclusion in the NLEM. Once a medicine is included into the NLEM, it can be reimbursed under all three public health insurance scheme. Contractual agreements like MEAs may be used as part of the negotiation process before making the final decision with the payer in Thailand.

## Discussion

This study summarises reimbursement practices for high-cost medicines and will be relevant to LMICs exploring the mechanisms to consider these options. The findings of the study revealed that the countries reviewed have established “alternative pathways” to reimburse high-cost medicines, as they are not cost-effective at the standard willingness-to-pay threshold. It also highlights several issues that need to be taken into account by countries when establishing mechanisms to consider such high-cost medicines for reimbursement.

These alternative pathways require particular eligibility criteria to be met such as rarity, severity, and the life-saving nature of medicine, before reimbursement is considered. However, it is important to define these criteria for a country’s population context to ensure its applicability. For example, lifesaving or extension of life criteria for a medicine is too ambiguous and if not defined clearly can lead to selective listing of medicines, thus impacting overall accessibility of the medicines. There is no clear guidance on the evidence required to predict the extension of life expectancy as a direct consequence of medicines being considered as there is uncertainty regarding treatment that has no alternative treatment options. Additionally, the criteria for clinical efficacy can be ambiguous and a sponsor may submit medicines with surrogate outcomes [26] and this can complicate the data collection for conditional agreements.

There have also been concerns around the management and sustainability of these programmes [18]. Previous critiques have focused on the prioritisation and allocation of funds such as the CDF, to only certain cancer patients, leading to questions about transparency and equity [27, 28]. Additionally, the lack of outcome data demonstrating actual benefits from funds like CDF is unknown, raising concerns surrounding their effectiveness [29]. Paradoxically, the existence of an alternative funding pathway like the CDF may reduce a manufacturer’s incentive to lower prices or invest in additional research to demonstrate cost-effectiveness [27]. Although there have been reforms to the CDF, there has been limited use of data in monitoring MEAs and the attendant uncertainties [30, 31].

While conditional agreements like MEAs have several known benefits, including flexibility in dealing with uncertainties for new and expensive medicines along with reported benefits of early access, concerns remain about their implementation and transparency. Several critics are also concerned about the general trend of using MEA as a quick fix or an ad hoc solution. Additionally, conditional agreement schemes in England have been criticised for failing to collect enough evidence to address outcome uncertainty [32]; [31] as well as for slowing access due to the complexity of agreements and resource-intensive activity prior to and after its administration (monitoring requirements, transaction costs, administrative costs) [22, 23, 33].

The study also shows the limitations of existing processes in countries to reimburse high-cost medicines. In Thailand, while medicines that are not cost-effective are typically excluded from the list, there have been a few exceptions, for example, such as imiglucerase and sofosbuvir which were included in the benefit package on account of equity considerations even though they did not provide good value-for-money [7]. Our study found that high-cost medicines are often not considered for submission. For example, it was found that almost a third of the 43 cancer medicines proposed were never submitted for consideration of the NLEM, given the view that only cost-effective medicines would be included in the final list. Thus, there is a selection bias in the medicines considered and those with a potentially high QALY loss would not enter the reimbursement process. This, on the one hand increases the efficiency of the system but on the other, limits the types of medicines that can be even considered for reimbursement.

The proposed framework seeks to apply the principle of HTA to reimburse any high-cost medicine that does not meet the cost-effectiveness criteria at the existing cost-effectiveness threshold value in Thailand. It has informed discussions on policy by the Sub-Committee for the Development of the Thai NLEM, which has decided to use the evidence on cost-effectiveness to reimburse high-cost medicines as a first step and make such medicines eligible for reimbursement. Thus, even as a medicine might not be deemed to be cost-effective, the information derived from the analysis, can facilitate the decision-making process of including such high-cost medicines through the NLEM mechanism and also potentially be used for negotiating the arrangements between the manufacturer and payer. In addition, the framework seeks to incorporate other relevant criteria such as, the life-saving or life-extending nature of the medicine, and the non-availability of alternative treatment options for any class of medicines by clearly defining them. As described above for these specific criteria, there may be need for making judgement calls in borderline cases of these definitions, for example, if medicine is able to extend life by 11 months it may require further deliberation. In line with international practices, however, a new sub-category “E3” was proposed with an upper limit on the cost-effectiveness threshold of THB 1–2 million as well as a budget ceiling of 5 million THB (USD 1.5 million). In terms of the implementation mechanism, the framework proposes the use of MEA after evaluating the cost-effectiveness and considering other criteria, essentially as tool for price negotiations as opposed to the common practice by industry to use MEA as an entry point for introducing high-cost medicines [34].

After this framework was agreed by the experts of Working Group commissioned for this study, in February 2023, the findings of this framework were further presented to relevant stakeholders of the Sub-Committee for the Development of Thai NLEM. After deliberations on the recommendations of this framework, informed a proposal for potential inclusion of such high-cost ineffective medicines if they meet specific criteria as mentioned above under the existing “E2” category. M&E has been planned for further improvement of this operational guideline.

There are limitations to this study. First, the study mainly reflects the Thai values and health infrastructure, it may therefore not be generalisable. Second, there are methodological challenges in incorporation of several uncertainty of high-cost medicines specially with novel targeted therapy, and the authors acknowledges that the traditional cost-effectiveness analysis might not address them all. However, with recent growing interest in the field of precision medicine and novel technologies, Thailand has set priority agendas and developing methodological guidelines for targeted therapy is one such item that is already been undertaken by HITAP [35]. Even if doing a typical cost-effectiveness analysis has methodological hurdles, by the time this framework is adopted completely, we should have a new reference case to use as a roadmap for conducting value-based assessments for novel, high-cost medicines. Lastly, the study draws its inspiration for framework from international experience reflecting existing processes in select countries rather than an evaluation of these processes, i.e. we know what the process looks like in Australia, England, South Korea, but we do not know how these processes meet the needs of their population and the constraint of the health budget. Also, since the selection of countries under review was based on convenience sampling it might not provide a complete picture of the international landscape.

In conclusion, high-cost medicines should only be reimbursed when they are cost-effective; and reimbursing those high-cost medicines that are not cost-effective as part of public health insurance schemes may be considered by countries when they are life-saving or do not have alternative treatment options. Context-specific factors will need to be applied and M&E will be critical to ensure successful implementation. HTA methods and processes are important in guiding these decisions to support sustainable access to affordable healthcare in pursuit of UHC.

### Supplementary Information

**Additional file 1: Table S1.** List of official websites of HTA agencies. **Table S2.** Definitions and special pathways in the reviewed countries. **Figure S1.** Survey results from the Working Group expert consultations. **Figure S2.** Comparative analysis of proposed 43 cancer drugs.

## Data Availability

The datasets used and/or analysed during the current study are available from the corresponding author on reasonable request.

## Abbreviations

ACE: Agency for Care Effectiveness
BCA: Benefit Criteria Advisory
CDF: Cancer Drug Fund
EE: Economic evaluation
EML: Essential medicine list
FDA: Food and Drug Administration
GBP: British Pound Sterling
HIRA: Health Insurance Review and Assessment Service
HITAP: Health Intervention and Technology Assessment Program
HST: Highly Specialised Technology
HTA: Health technology assessment
IBEP: Introduction of the Benefit Enhancement Plan
ICER: Incremental Cost-Effectiveness Ratio
IMF: Innovative Medicines Fund
LMIC: Low- and middle-income countries
LSDP: Life Saving Drug Program
MAA: Managed Access Agreements
*MEA*: Managed Entry Agreements
MOHW: Ministry of Health and Welfare
NECA: National Evidence-based Healthcare Collaborating Agency
NHIS: National Health Insurance Service
NHS: National Health Service
NHSO: National Health Security Office
NICE: National Institute of Care and Excellence
NLEM: National List of Essential Medicine
NLEV: National List of Essential Vaccine
PAS: Patient Access Scheme
PBAC: Pharmaceutical Benefit Advisory Committee
PBC: Pharmaceutical Benefit Committee
PBS: Pharmaceutical Benefit Scheme
PE: Pharmaco-economics
QALY: Quality-Adjusted Life Year
RSA: Risk sharing agreement
THB: Thai Bhat
UCBP: Universal Care Benefit package
UCS: Universal Care Scheme
UHC: Universal Health Coverage
USD: United States Dollar
WHO: World Health Organization
WTP: Willingness to pay
UK: United Kingdom
