# Supplementary material for: Expanding access to high-cost medicines under the Universal Health Coverage scheme in Thailand: review of current practices and recommendations
Source: J Pharm Policy Pract. 2023 Nov 7;16:138. doi: 10.1186/s40545-023-00643-z (PMC10631213; doi:10.1186/s40545-023-00643-z)

Additional file 1: Review of international landscape

Table S1: List of official websites of HTA agencies

| **Countries** | **Link** |
| --- | --- |
| Australia | <https://pbac.pbs.gov.au/> |
| Canada | CADTH: <https://www.cadth.ca/> |
| Malaysia | MaHTAS: <https://www.moh.gov.my/> |
| Singapore | <https://www.ace-hta.gov.sg/about-us> |
| South Korea | <https://www.hira.or.kr/eng/about/05/02/06/index.html> |
| UK | NICE: <https://www.nice.org.uk/>  NHS: <https://www.england.nhs.uk/cancer/cdf/> |

Table S2: Definitions and special pathways in the reviewed countries

The stakeholders were contacted through direct email, and discussions took place from May through July 2022. Given the time constraints, the research team utilised its existing network to recruit experts through convenience sampling. Six experts recruited were health economists with extensive knowledge or working experience with reimbursements in select countries as follows: Canada’s Drug and Health Technology Agency (CADTH), Malaysian Health Technology Assessment Section (MaHTAS), University of York England, Republic of Korea’s National Evidence-Based Healthcare Collaborating Agency (NECA), Agency for Care Effectiveness (ACE), Singapore and South Australian University, Australia.

|  | **Countries** | | | | | | |
| --- | --- | --- | --- | --- | --- | --- | --- |
|  | **Thailand** | **England** | **Malaysia** | **Australia** | **South Korea** | **Canada** | **Singapore** |
| **Definition** | | | | | | | |
| Does the country have specific definition for high-cost medicine? | - | √ | - | - | - | - | - |
| Does the country have specific definition for rare disease? | √ | √ | - | √ | √ | √ | √ |
| Does the country have special list of cancer medicines? | √ | √ | - | √ | √ | √ | √ |
| Is there a high cost-effectiveness threshold for rare, cancer or other medicines? | - | √ | - | - | - | - | - |
| **Special Pathways** | | | | | | | |
| Does the country have a special pathway to reimburse high-cost medicine? | - | √ | - | √ | √ | √ | - |
| Does the country have a special pathway for reimbursing rare disease medicines? | √ | √ | √ | √ | √ | √ | √ |
| Does the country have a special pathway for reimbursing cancer medicines? | - | √ | √ | √ | √ | √ | √ |
| **Implementation** | | | | | | | |
| Does the country implement MEAs? | √ | √ | √ | √ | √ | √ | √ |
| Is there process guide for implementation? | - | √ | - | √ | √ | - | - |
| **Monitoring & Evaluation** | | | | | | | |
| Is there M&E of special pathways? | - | √ | - | √ | √ | - | - |
| Is there a defined time to review special pathways? | - | √ | - | √ | √ | - | - |
| Any recommendations adapted? | - | √ | - | √ | - | - | - |

Figure S1: Survey results from the Working Group expert consultations


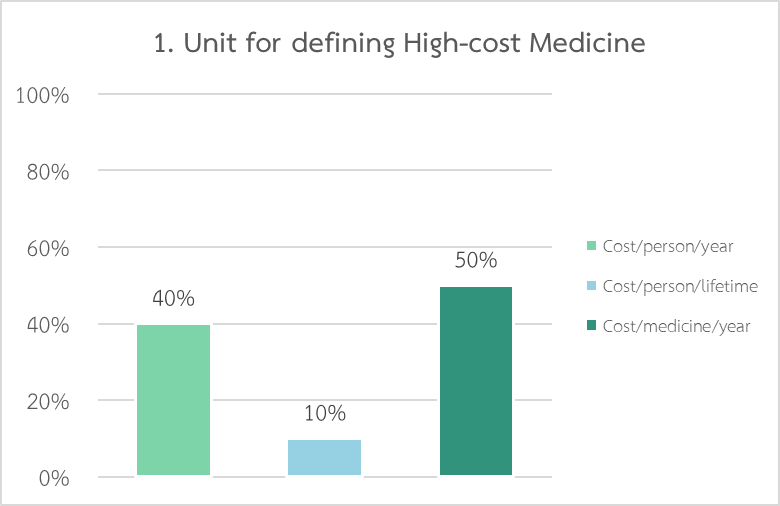


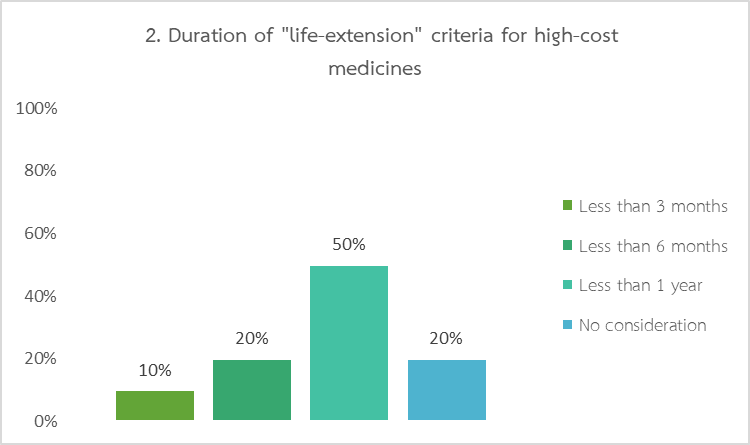

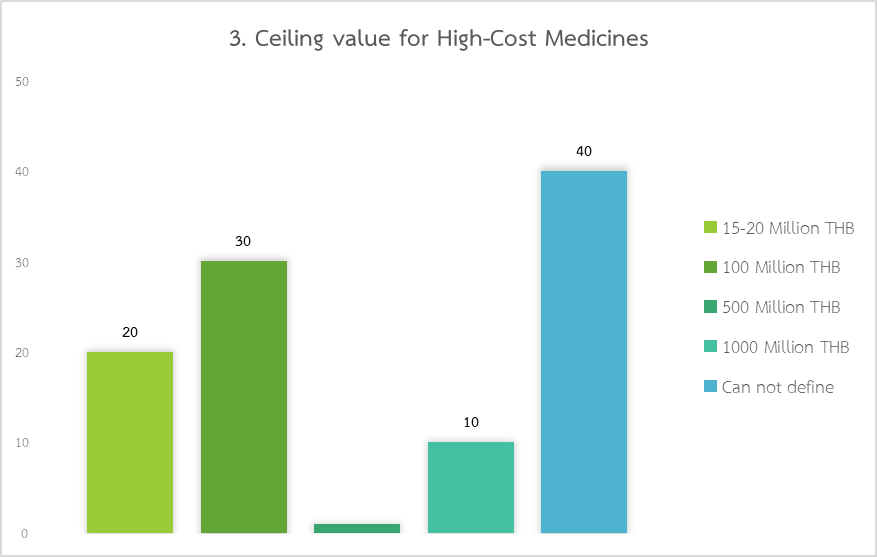


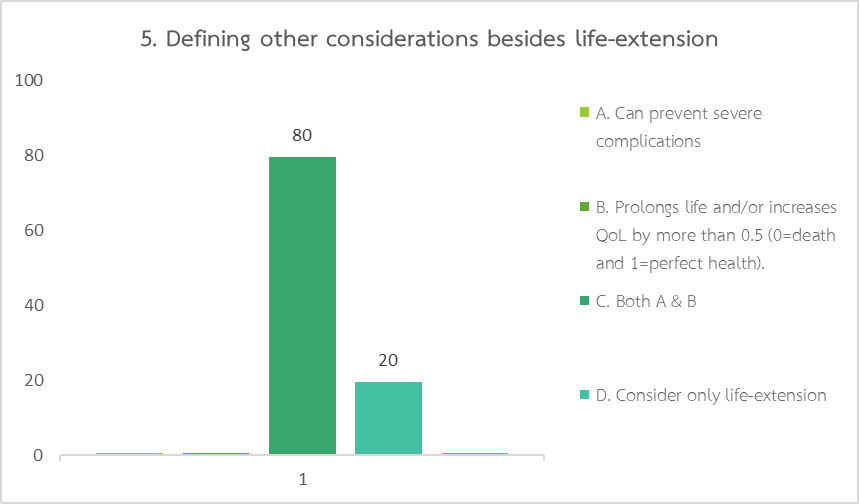

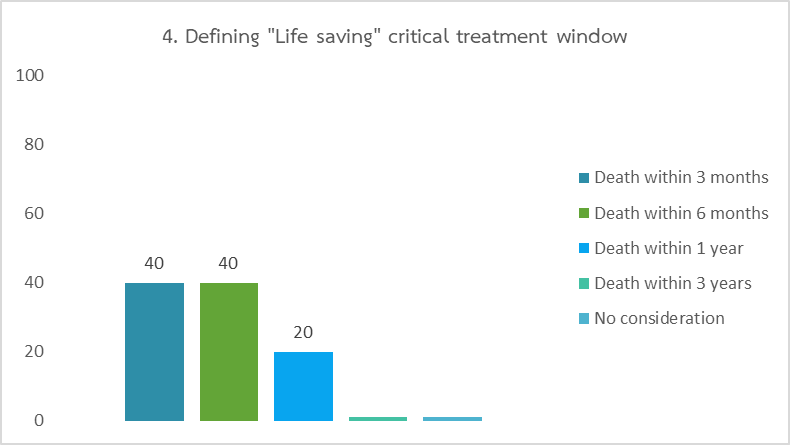


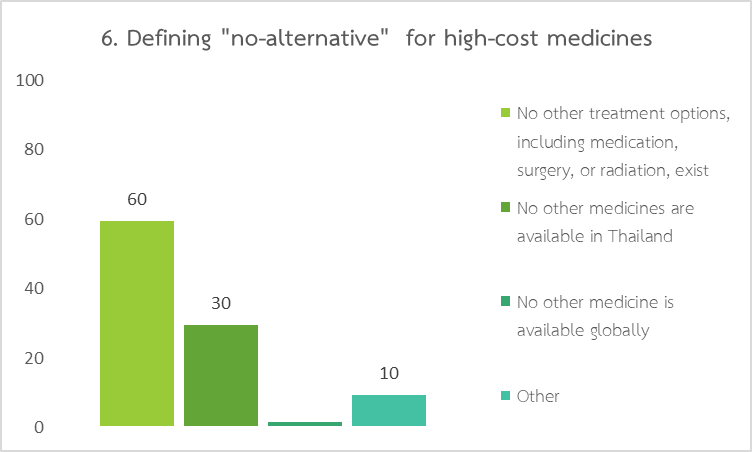


Figure S2: Comparative analysis of proposed 43 cancer drugs.


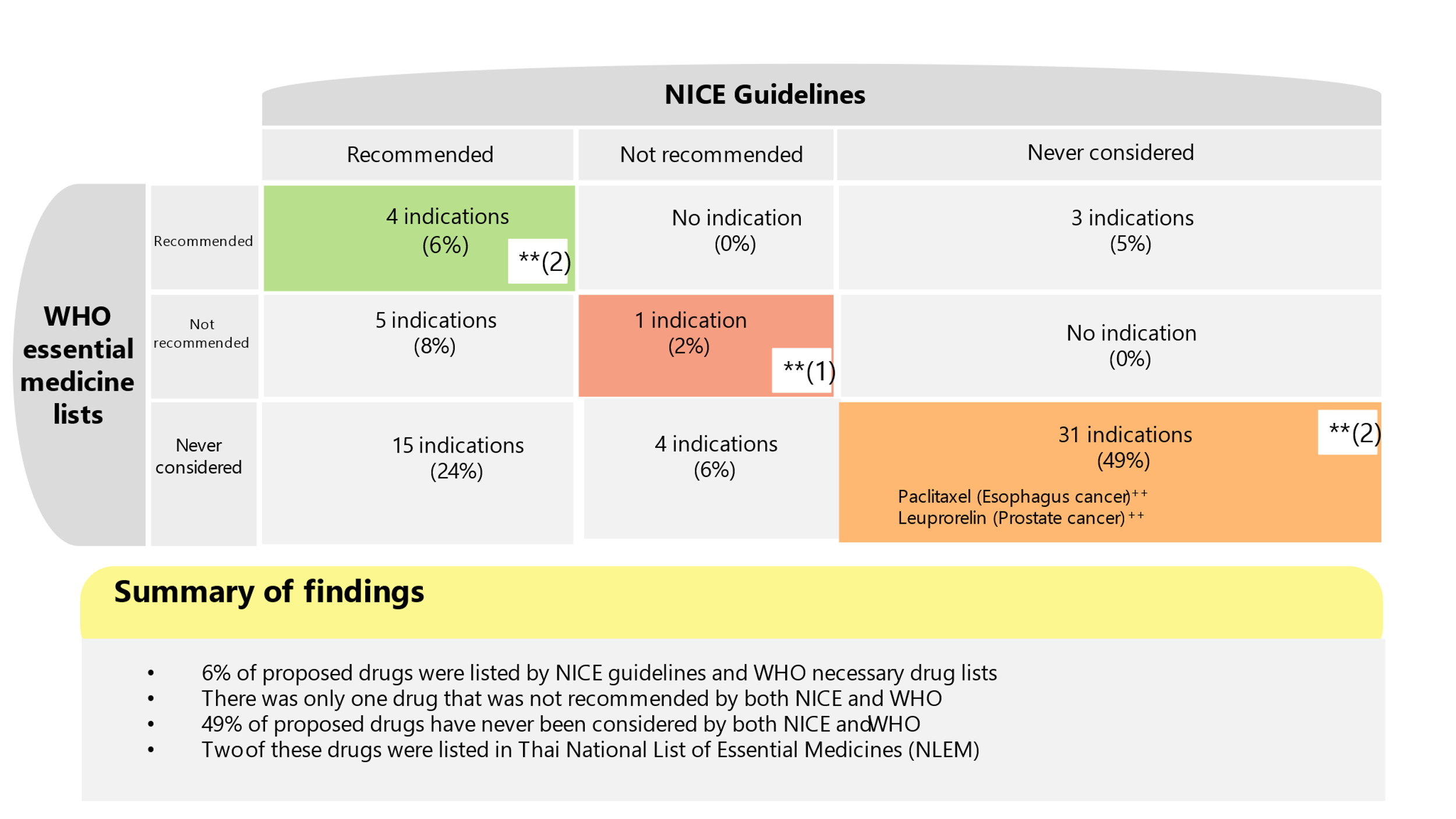

Supplement: Supplementary file 1 — Additional file 1: Table S1. List of official websites of HTA agencies. Table S2. Definitions and special pathways in the reviewed countries. Figure S1. Survey results from the Working Group expert consultations. Figure S2. Comparative analysis of proposed 43 cancer drugs. [file 40545_2023_643_MOESM1_ESM.docx]
